# Mathematical explanation of the predictive power of the X-level approach reaction noise estimator method

**DOI:** 10.1186/1742-4682-9-12

**Published:** 2012-04-13

**Authors:** Zoran Konkoli

**Affiliations:** 1 Chalmers University of Technology, Gothenburg, Sweden

## Abstract

The X-level Approach Reaction Noise Estimator (XARNES) method has been developed previously to study reaction noise in well mixed reaction volumes. The method is a typical moment closure method and it works by closing the infinite hierarchy of equations that describe moments of the particle number distribution function. This is done by using correlation forms which describe correlation effects in a strict mathematical way. The variable X is used to specify which correlation effects (forms) are included in the description. Previously, it was argued, in a rather informal way, that the method should work well in situations where the particle number distribution function is Poisson-like. Numerical tests confirmed this. It was shown that the predictive power of the method increases, i.e. the agreement between the theory and simulations improves, if X is increased. In here, these features of the method are explained by using rigorous mathematical reasoning. Three derivative matching theoremsare proven which show that the observed numerical behavior is generic to the method.

## Introduction

Noise is an integral part of the workings of the living cell biochemistry [1]. There are many types of noise and this work focuses on the intrinsic noise. If reactant copy numbers are low they can fluctuate widely [2]. These fluctuations can severely influence the dynamics of the cell and need to be carefully controlled [3].

Describing intrinsic noise has attracted a lot of effort. A range of theoretical methods have been developed to study intrinsic noise. However, an accurate characterization of the reaction noise is not easy. A direct solution of the chemical master equation for the system is often not possible since the number of configurations can be exponentially large. Numerical simulation methods can be used to avoid this problem, and are often implemented by using the Gillespie algorithm [4]. However, to obtain accurate prediction for moments of the particle number distribution function, e.g. the variance, sampling with a relatively large number of runs (simulations) is needed. This becomes impractical if the number of particle types is very large. A range of methods have been suggested to complement or replace these techniques. The focus of this work is on moment closure techniques [5-15].

The main idea behind moment closure approaches is to construct the equation system that can describe various moments of the particle number distribution function. In such a way there is no need to directly solve the master equation or perform a largenumber of computer simulations. The problem is that the equation system that describes these moments is, in principle, infinite. The main issue is to cut (or close) the hierarchy. This is done in two ways. First, one can try to make some specific assumptions about the reacting system which can be used to express higher order moments in terms of lower order ones. This procedure defines the moment closure function for the problem. Second possibility is to take the distribution function centered approach and assume that the particle number distribution function has a well-defined form, parameterized by a finite set of parameters. Variational calculus can be used to obtain the parameters [7,16]. In both cases the complicated many body dynamics is reduced to a set of ordinary differential equations that govern quantities of interest.

Two moment closure methods that were suggested previously will be of a particular interest. The PARNES method [14,15] is based on the assumption that pair effects dominate the dynamics. The method has been generalized into the XARNES method [17] so that higher order correlation effects can be included in the description. In fact, various choices for X result in a series of methods, e.g. X = P (pair effects), T (triplets), Q (quadruples), etc. Thus the PARNES method is the special case of the XARNES method with X = P. Both methods are based on the rather generic formalism of correlation forms which is used in statistical physics to model spatially extended many body effects. Each correlation form describes a particular correlation effect (single, pair, triple, and so forth). Correlation forms are used to perform a cluster like expansion of relevant quantities of interest. In the previous studies, the original correlation form formalism [18,19] used to model spatially extended diffusion controlled reactions has been adapted for describing well mixed reaction volumes.

A moment closure method works well in the intended domain of application, but it might easily fail if used elsewhere. Thus for a moment closure method to be useful it is necessary to, if possible, specify precisely in which situations the method is expected to work well. In [17] it was argued that the XARNES method should describe well systems with a Poisson-like particle number distribution function. This was confirmed by numerical studies. In here, it will be shown rigorously that such behavior is generic to the method. Also, there are some ambiguities while adapting the correlation form formalism to the well mixed situation, and the procedure is not unique. The problem is that a large number of spatial degrees offreedom need to be projected onto a much small number of variables, and there are many ways how this can be done. In here, it will be shown that the choice made in the previous studies is in some sense optimal.

To show all of the above the derivative matching procedure introduced in [9-11] will be used. In particular, the procedure from the technical report [9] willbe closely followed. This report was formalized in much shorter form in [10]. A very general multiplicative ansatz was suggested for the moment closure function. The precise form of the ansatz was found by using the derivative matching procedure. The function was parameterized by a finite set of parameters which were found by matching time derivatives of exact and approximate moments. This was done for the system in the pure state. The condition for a good match was derived in the form of a generic formula that involves the moment closure function parameters.

The same formula was obtained in a different context where the XARNES method was suggested [17]. This fact motivated the present work. The derivative matching procedure will be applied to investigate the accuracy of the XARNES method. There are some important differences in the setups in [10,11] and the present work. In here, the original procedure from [10,11] will be carried in the opposite direction. The XARNES method is based on the well-defined multiplicative ansatz for factorial moments. It will be shown that the ansatz implies good derivative matching properties. Also, while the original work focused on the pure state in here the Poisson state will be of interest.

## The mathematical setup

A reaction system is defined as follows. It is assumed that reacting particles mix well and that particle positions are irrelevant. A configuration of the system can be specified by listing how many particles of each type there are in the system

(1) $$\vec{n}=\left(n_{1},n_{2},\cdots \;,n_{i},\cdots \;,n_{T}\right)$$

where variables *n_i_*, *i *= 1, 2,..., *T*, are positive definite integers used to denote the particle numbers. A configuration of the system changes in time due to the presence of reactions.

The full list of reactions is given by *r*_1_, *r*_2_,..., *r_R _*and a reaction r_α _is formally defined in the usual chemical notation as

(2) $$u_{\alpha 1}X_{1}+\ldots +u_{\alpha T}X_{T}\overset{\lambda_{\alpha }}{\to }v_{\alpha 1}X_{1}+\ldots +v_{\alpha T}X_{T}$$

The positive definite vectors

(3) $${\vec{u}}_{\alpha }=\left(u_{\alpha 1},u_{\alpha 2},\cdots \;,u_{\alpha R}\right)$$

(4) $${\vec{v}}_{\alpha }=\left(v_{\alpha 1},v_{\alpha 2},\cdots \;,v_{\alpha R}\right)$$

with α = 1,2,..., *R *contain the stoichiometry coefficients for the reactions. It is assumed that the dynamics can be modeled as the Markov process where λ_α _is the reaction rate for a reaction r*_α _*having the unit ofinverse time. The dynamics defined in such a way is stochastic.

One possible way to describe the dynamics and characterize noise is to construct the master equation, solve it, and obtain the particle number distribution function $P\left(\vec{n},t\right)$ which describes all stochastic properties of the system. However, both the computation and the direct inspection of the particle number distribution function is often tedious. It is more useful to investigate certain properties of the distribution function.

In practice this is done by computing various observables, or ensemble averages, as

(5) $$\left\langle f\left(\vec{n}\right)\right\rangle \equiv \underset{\vec{n}}{\sum }f\left(\vec{n}\right)P\left(\vec{n},t\right)$$

where *f *is the function that suitably parameterizes an observable of interest. A typical observable could be some low level moment such as the mean or the variance. Clearly, the direct use of (5) is not practical for large systems and one needs to avoid this route somehow. The idea is to project the details of the dynamics and obtain a coarse grained description by monitoring a selected set of observables instead of all configurations. These observables will be classified, i.e. labeled, in a precise mathematical way. For a fixed number of particle types, it is useful to introduce the vector space of all admissible labels. Formally, this is done through the following set of definitions.

**Definition 1**. The space of vectors that are used to label various correlation effects or, depending on the context, observables, will be denoted by Ω,

(6) $$\Omega \equiv \left\{\vec{m}|m_{i}\geq 0,i=1,2,\cdots \;,T\right\}$$

where a variable *m_i _*is a positive definite integer. For example, the average number of particles of a type *i *will be labeled as ${\vec{e}}_{i}=\left(0,0,\cdots \;,0,1,0,\cdots \;,0\right)$ where the digit 1 appears on the i-the place.

**Definition 2**. It is useful to introduce the order, or norm, of a vector $\vec{m}\in \Omega$ as the sum of its components,

(7) $$∥\vec{m}∥=\overset{T}{\underset{i=1}{\sum }}m_{i}$$

This order will be used to classify various correlation effects. It is clear from the definition that

(8) $$∥{\vec{m}}_{1}+{\vec{m}}_{2}∥=∥{\vec{m}}_{1}∥+∥{\vec{m}}_{2}∥$$

The full set of observables will be split into two disjoint subsets. The first subset contains the observables that are being included in the projection and the second set contains the observables that are omitted. The observables in the second set need to be expressed somehow in terms of the observables in the first set. The following definitions will be used to classify such sets.

**Definition 3**. The set of vectors $\vec{m}\in \Omega$ that contains all vectors with orders 0,1,2,...,ξ will be denoted by Ω_ξ_,

(9) $$\Omega_{\xi }=\left\{\vec{m}\in \Omega |\;||\vec{m}||\;\leq \xi \right\}$$

where *ξ *≥ 1 is an arbitrary integer used to classify various theories.

**Definition 4**. In a similar way,

(10) $$\Omega_{\vec{m}}=\left\{{\vec{m}}^{\prime }\in \Omega |m_{1}^{\prime }\leq m_{1},m_{2}^{\prime }\leq m_{2},\cdots \;,m_{T}^{\prime }\leq m_{T}\right\}$$

will denote the set of vectors ${\vec{m}}^{\prime }\in \Omega$ that are smaller than a vector $\vec{m}$ in the lexical sense defined above.

The set Ω**_ξ _**will be used to denote the set of observables being explicitly considered in the theory. For example, the mean field theory (the classical chemical kinetics) that neglect effects of fluctuation works with the first order effects where ξ = 1 (X = Singles). Thus the mean field theory would work by constructing equations for all $\rho_{\vec{e}i}$ with ${\vec{e}}_{i\in \Omega_{1}}$. Sets of the type $\Omega_{\vec{m}}$ will be used to limit various sums.

**Definition 5**. Finally, the set of all vectors that are not in Ω***_ξ _***will be denoted as,

(11) $${\bar{\Omega }}_{\xi }=\Omega \backslash \Omega_{\xi }$$

A vector from this set will be denoted by the bar character above the vector symbol, e.g. $\bar{m}\in {\bar{\Omega }}_{\xi }$.

Further, several definitions will prove useful that generalize well known operations on numbers to similar operations in Ω. These generalizations greatly compactify some of the mathematical expressions that will be discussed.

**Definition 6**. Let ${\vec{\omega }}_{1}$ and ${\vec{\omega }}_{2}$ be two arbitrary tuples of real numbers with rank *T*, such as $\vec{\omega }=\left(\omega_{1},\omega_{2},\cdots \;,\omega_{T}\right)$. The set of all such vectors will be denoted by *R^T^*. For any pair of such vectors the generalized power will be defined as

(12) $${\vec{\omega }}_{1}^{{\vec{\omega }}_{2}}\equiv \omega_{11}^{\omega_{21}}\omega_{12}^{\omega_{22}}\omega_{13}^{\omega_{23}}\cdots \omega_{1i}^{\omega_{2i}}\cdots \omega_{1T}^{\omega_{2T}}$$

Obviously, Ω⊂*R^T ^*and this definition can be also used for vectors in Ω. It is clear from the definition that

(13) $${\vec{\omega }}^{{\vec{\omega }}_{1}+{\vec{\omega }}_{2}}={\vec{\omega }}^{{\vec{\omega }}_{1}}{\vec{\omega }}^{{\vec{\omega }}_{2}}$$

**Definition 7**. The binomial like coefficient that involves a pair of vectors ${\vec{m}}_{1}$ and ${\vec{m}}_{2}$ from Ω is formally defined as

(14) $$\left(\begin{array}{c} {\vec{m}}_{1}\\ {\vec{m}}_{2} \end{array}\right)\equiv \left(\begin{array}{c} m_{11}\\ m_{21} \end{array}\right)\left(\begin{array}{c} m_{12}\\ m_{22} \end{array}\right)\left(\begin{array}{c} m_{13}\\ m_{23} \end{array}\right)\cdots \left(\begin{array}{c} m_{1i}\\ m_{2i} \end{array}\right)\cdots \left(\begin{array}{c} m_{1T}\\ m_{2T} \end{array}\right)$$

and it is assumed that a binomial coefficient $\left(\begin{array}{c} m_{1i}\\ m_{2i} \end{array}\right)$ in the product is zero if *m*_2*i *_>*m*_1*i*_.

**Definition 8**. The factorial-like symbol applied to a vector $\vec{m}$ from Ω is generalized as

(15) $$\vec{m}!\equiv m_{1}!m_{2}!m_{3}!\cdots m_{i}!\cdots m_{T}!$$

**Definition 9**. The product between two real numbers is generalized as

(16) $${\vec{\omega }}_{1}⊙{\vec{\omega }}_{2}=\left(\omega_{11}\omega_{21},\cdots \;,\omega_{1i}\omega_{2i},\cdots \;,\omega_{11}\omega_{21},\right)$$

where ${\vec{\omega }}_{1}$ and ${\vec{\omega }}_{2}$ are two arbitrary vectors from *R^T^*. Please note that the result of the operation is an element in the same set, ${\vec{\omega }}_{1}⊙{\vec{\omega }}_{2}\in R^{T}$. Also, the following identity related to this definition will be useful later on,

(17) $${\left({\vec{\omega }}_{1}⊙{\vec{\omega }}_{2}\right)}^{\vec{m}}={\vec{\omega }}_{1}^{\vec{m}}{\vec{\omega }}_{2}^{\vec{m}}$$

## Exact equations of motion

There are several types of observables one could choose to work with. In this work factorial moments will be used. A factorial moment will be labeled by the related positive definite

vector $\vec{m}=\left(m_{1},m_{2},\cdots \;,m_{T}\right)\in \Omega$ and is defined as

(18) $$p\vec{m}=\langle \left(\begin{array}{c} \vec{n}\\ \vec{m} \end{array}\right)\vec{m}!\rangle$$

It was shown in [17] that the equation system for the exact factorial moments is given by

(19) $$\frac{d}{dt}p_{\vec{m}}\left(t\right)=\overset{R}{\underset{\alpha =1}{\sum }}\lambda_{\alpha }\underset{\vec{c}\in \Omega_{R}}{\sum }\left(\begin{array}{c} \vec{m}\\ \vec{c} \end{array}\right)\Gamma_{\alpha }\left(\vec{c}\right)\rho_{\vec{m}-\vec{c}+{\vec{u}}_{\alpha }}\left(t\right)$$

and the structure of the equations will be explained in the following. Γ coefficients in the sum are given by

(20) $$\Gamma_{\alpha }\left(\vec{c}\right)=\left[\left(\begin{array}{c} {\vec{v}}_{\alpha }\\ \vec{c} \end{array}\right)-\left(\begin{array}{c} {\vec{u}}_{\alpha }\\ \vec{c} \end{array}\right)\right]\frac{\vec{c}!}{{\vec{u}}_{\alpha }!}$$

for all $\vec{m}\in \Omega$. A vector $\vec{c}\in \Omega_{R}$ is a positive definite vector to be referred to as a contraction vector. Contraction vectors emerge during the field theoretic derivation of the equations of motion when one uses the Wick theorem. More details regarding the field theoretic setup can be found in [14,15]. One can derive the same set of equations in another way (not shown). Sums over contraction vectors will be of the central interest in the following.

The space of all possible contractions is defined by reactions that can occur in the system.

From the definition of Γ coefficients in (20) one can see that for a fixed ${\vec{u}}_{\alpha }$ or ${\vec{v}}_{\alpha }$ the sum over the contraction vectors in (19) is restricted.

This is suggestive of the following formal definition of the space Ω*_R_*⊂Ω:

(21) $$\Omega_{R}={⋃}_{\alpha =1}^{R}\left(\Omega_{{\vec{u}}_{\alpha }}⋃\Omega_{{\vec{v}}_{\alpha }}\right)$$

Despite the fact that the sum over contraction vectors is finite, the equation system for exact moments represents, in principle, an infinite hierarchy of equations since on the right hand side of the equation for a given $\rho_{\vec{m}}$ there are terms that involve higher order moments since it may happen that $∥\vec{m}-\vec{c}+{\vec{u}}_{\alpha }∥>∥\vec{m}∥$. The hierarchy of equations for the exact moments appears not particularly useful. However, it can be used to devise approximation schemes. If higher order moments can be expressed in terms of few lower order ones then the equation system closes down.

## A way of closing the hierarchy: the XARNES method

The XARNES method is based on the closure ansatz which is constructed by using the concept of correlation forms [14,15,17]:

(22) $$\ln\;\nu_{\vec{m}}\left(t\right)=\underset{\hat{m}\in \Omega \xi }{\sum }\left(\begin{array}{c} \vec{m}\\ \hat{m} \end{array}\right)w_{\hat{m}}\left(t\right)$$

where $w_{\hat{m}}$ denotes the correlation form labeled by a vector $\hat{m}\in \Omega$. The detailed motivation behind this equation is given in [17]. It is assumed that that correlation forms with the orders above a given threshold *ξ *can be assumed small, $w_{\vec{m}}\left(t\right)\approx 0\Leftrightarrow ∥\vec{m}∥>\xi$. Since a moment $\nu_{\vec{m}\left(t\right)}$ is not exactly equal to the related moment $\rho_{\vec{m}\left(t\right)}$, two different symbols, *v *and *ρ*, are used to denote them. However, if the approximation above works well, possibly when the number of vectors in Ω_ξ _becomes large, their values should be close.

Please note that if a factorial moment is zero, the left hand side of Eq. (22) becomes infinite owing to the singularity of the logarithmic function. This implies that the related correlation form becomes also infinite. Clearly, by construction, the XARNES ansatz is somewhat ambiguous for cases when some factorial moments vanish. In what follows it will be assumed that all factorial moments are strictly larger than zero but they can be arbitrary close to zero.

It was shown previously [14,15,17] that the assumption (22) is an implicit ansatz for defining the function that expresses a higher order non-base factorial moments $\nu_{\bar{m}\left(t\right)}$ with $\bar{m}\in {\bar{\Omega }}_{\xi }$ in terms of the base moments $\vec{\nu }\left(t\right)\equiv (\cdots \;,{\nu_{\hat{m}\left(t\right),\cdots }}_{)}$ with $\hat{m}\in \Omega_{\xi }$:

(23) $$v_{\bar{m}}\left(t\right)=\psi_{\bar{m}}\left(\vec{\nu }\left(t\right)\right);\bar{m}\in {\bar{\Omega }}_{\xi }$$

where the moment closure function is given by

(24) $$\psi_{\bar{m}}\left(\vec{\nu }\left(t\right)\right)=\underset{\hat{m}\in \Omega_{\xi }}{\prod }v_{\hat{m}}{\left(t\right)}^{\gamma_{\hat{m}}^{\bar{m}}}$$

and *γ *coefficients are defined as

(25) $$\gamma_{\hat{m}}^{\bar{m}}=\underset{{\hat{m}}^{\prime }\in \Omega_{\xi }}{\sum }\left(\begin{array}{c} \bar{m}\\ {\hat{m}}^{\prime } \end{array}\right){\left(C^{-1}\right)}_{{\hat{m}}^{\prime },\hat{m}}$$

with the matrix *C *specified as

(26) $$C_{{\hat{m}}_{1},{\hat{m}}_{2}}=\left(\begin{array}{c} {\hat{m}}_{1}\\ {\hat{m}}_{2} \end{array}\right)$$

Also, by construction one has that $\psi_{\hat{m}}\left(\vec{\nu }\left(t\right)\right)=v_{\hat{m}}\left(t\right)$.

Finally, by combining (24) with (19) gives the XARNES system of equations,

(27) $$\frac{d}{dt}\nu_{\hat{m}}\left(t\right)=\overset{R}{\underset{\alpha =1}{\sum }}\lambda_{\alpha }\underset{\vec{c}\in \Omega_{R}}{\sum }\left(\begin{array}{c} \hat{m}\\ \vec{c} \end{array}\right)\Gamma_{\alpha }\left(\vec{c}\right)\psi_{\hat{m}-\vec{c}+\vec{u}\alpha }\left(\vec{\nu }\left(t\right)\right)$$

for $\hat{m}\in \Omega_{\xi }$.

It is clear from the form of equation (19) that $\rho_{\vec{0}}=\left\langle 1\right\rangle$ does not depend on time. For $\vec{m}=0$ the sum over contraction vectors can only contain one vector, $\vec{c}=\vec{0}$, and since $\Gamma_{\alpha }\vec{0}=0$ the time derivative of $\rho_{\vec{0}}$ vanishes. This expresses the fundamental probability conservation requirement for any reasonable theory. In contrast to the $\rho_{\vec{0}}$ moment, a correlation moment with $\vec{m}\neq 0$ is a real dynamic quantity. Based on this one might partition the Ω**_ξ _**space in two spaces. The first space should consist of the null vector, while the second space would consist of all other vectors in Ω**_ξ_**. It is possible to show that the values of *γ *are same regardless whether the zero vector is singled out or not.

## An intriguing similarity

A similar set of equations as the one given by (24-26) has been obtained in a slightly different context [10,11] where the multiplicative ansatz given in (24) was the starting point in developing a moment closure method for zero centered moments, $\eta_{\vec{m}}$, which in the notation of the present work would be given by

(28) $$\eta_{\vec{m}}\equiv \left\langle {\vec{n}}^{\vec{m}}\right\rangle$$

The goal was to determine the values of the *γ *coefficients in Eq (24) from the requirement that time derivatives of exact and approximate moments match at the time instance when the distribution function resembles the distribution function of the pure system. It was shown that the derivatives match best if the coefficients *γ *are given exactly by (25). Thus the equations would be identical if not for the fact that the related equations in [10,11] are for zero-centered moments.

This remarkable coincidence where the same set of coefficients is obtained in two different ways is rather intriguing. In particular, this strongly suggests that the XARNES method might have advantageous properties as it comes to the derivative matching between the exact and the approximate moments computed for a suitable initial condition. It will be shown by employing a strict mathematical analysis, in the same way as done in [10,11], that this is indeed the case for the Poisson initial condition. This in turn explains the previous numerical observation from [14,15,17] that the XARNES method performs better when the particle number distribution function is Poisson-like as compared to the situation when the distribution resembles the one of the pure system.

## Derivative matching setup

In here a similar procedure as in [10,11] will be carried out to show that the XARNES ansatz expressed in (24) and (25) has some advantageous derivative matching properties. The original procedure will be implemented as follows. Consider the Eqs. (19) and(27) at time *t = t*_0 _where the particle number distribution function is strictly given by the uncorrelated multivariate Poisson distribution. In such a case all correlation forms are zero except the ones specified by vectors ${\vec{e}}_{i},i=1,\ldots ,T$. Thus the multivariate (uncorrelated) Poisson distribution is parameterized by parameters $\vec{\mu }=\left(\mu_{1},\mu_{2},\cdots \;,\mu_{T}\right)\in R^{T}$ where $\mu_{i}=\exp(w_{\vec{e}i)}$. By a trivial application of Eq. (22) one can see that the exact factorial moments of the reacting system computed at *t = t*_0 _are given by

(29) $$\rho_{\vec{m}}\left(t_{0}\right)\equiv {\vec{\mu }}^{\vec{m}}$$

for any $\vec{m}\in \Omega$.

At *t = t*_0 _the base factorial moments that are used in the XARNES method have to be chosen. This choice specifies the boundary condition for the XARNES equation of motion in (27). Naturally, for the purposes of comparing time derivatives it will be assumed that

(30) $$v_{\hat{m}}\left(t_{0}\right)=\rho_{\hat{m}}\left(t_{0}\right)={\vec{\mu }}^{\vec{m}};\hat{m}\in \Omega \xi$$

since if the values of the exact and the approximate base moments do not match their derivatives will not likely match either. The question is: given that the values of the base and the exact moments are same, can one expect that time derivatives of these quantities match as well?

In order to make some progress in answering this question a couple of identities involving *γ *coefficients will be needed. These identities are hard to prove by using the explicit form of these coefficients given in (25). The formalism of generating functions will be used instead. Before doing that the following two definitions need to be stated,

**Definition 10**. Symbol $P_{\varepsilon }\left(\vec{\omega }\right)$ will be used to denote a polynomial

(31) $$P_{\varepsilon }\left(\vec{\omega }\right)=\overset{∥\vec{m}∥\geq \varepsilon }{\underset{\vec{m}\in \Omega }{\sum }}A_{\vec{m}}{\vec{\omega }}^{\vec{m}}$$

where $A_{\vec{m}}$ are arbitrary real valued coefficients. No other restrictions are imposed on the sum.

**Definition 11**. Let $P_{\varepsilon }\left(\vec{\omega }\right)$ be a polynomial as defined previously. Symbol $\left[{\vec{\omega }}^{\vec{m}}\right]$ will be used to denote the operator that extracts the coefficient in front of a particular ${\vec{\omega }}^{\vec{m}}$ term in the polynomial, i.e. $A_{\vec{m}}$:

(32) $$\left[{\vec{\omega }}^{\vec{m}0}\right]\underset{\vec{m}\in \Omega_{*}}{\sum }A_{\vec{m}}{\vec{\omega }}^{\vec{m}}=\left\{A_{\begin{array}{cc} {}_{{\vec{m}}_{0}} & {\vec{m}}_{0}\in \Omega *\\ 0 & {\vec{m}}_{0}\notin \Omega * \end{array}}\right)$$

for any Ω_* _⊂ Ω. Likewise,

(33) $$\left[{\vec{\omega }}^{{\vec{m}}_{0}}\right]P_{\varepsilon }\left(\vec{\omega }\right)=\left\{A_{\begin{array}{c} {\vec{m}}_{0}\\ 0 \end{array}}\begin{array}{c} ∥{\vec{m}}_{0}∥\geq \varepsilon \\ ∥{\vec{m}}_{0}∥<\varepsilon \end{array}\right)$$

The following lemma is extremely useful for proving a couple of identities that will be needed later. The lemma has the form of a generating function identity.

**Lemma 1**. Let coefficients ***γ ***be defined by Eqs. (22) and (35). In other words, the only requirement imposed on these coefficients is that they are used to parameterize higher order factorial moments in terms of the base moments with the XARNES ansatz implied. In such a case these coefficients obey the following identity

(34) $$\begin{array}{c} \underset{\hat{m}\in \Omega_{\xi }}{\sum }\gamma_{\hat{m}}^{\bar{m}}{\left(1+\vec{\omega }\right)}^{\hat{m}}=\\ {\left(1+\vec{\omega }\right)}^{\bar{m}}+P_{\xi +1}\left(\vec{\omega }\right) \end{array}$$

where $\vec{\omega }$ and 1 ≡ (1,1,...,1) are vectors in *R^T ^*and $\bar{m}\in {\bar{\Omega }}_{\xi }$. Eq. (34) will be referred to as the generating function equation.

*Proof*. This identity can be proven as follows. First, one combines the XARNES ansatz (24) rewritten as

(35) $$\ln\;\nu_{\bar{m}}=\underset{\hat{m}\in \Omega \xi }{\sum }\gamma_{\hat{m}}^{\bar{m}}\ln\;\nu_{\hat{m}}$$

with the definition of the correlation forms (22) to obtain

(36) $$\underset{\hat{m}\in \Omega \xi }{\sum }\gamma_{\hat{m}}^{\bar{m}}\ln\;\nu \hat{m}=\underset{\hat{m}\in \Omega \xi }{\sum }\left(\begin{array}{c} \vec{m}\\ \hat{m} \end{array}\right)w_{\hat{m}}$$

By assuming that all correlation forms have predefined values given by

(37) $$w_{\hat{m}}={\vec{\omega }}^{m}$$

where $\vec{\omega }\in R^{T}$ is arbitrary but fixed, leads to the equation that is central for the proof of the lemma,

(38) $$\underset{\hat{m}\in \Omega \xi }{\sum }\gamma_{\hat{m}}^{\bar{m}}\ln\;\nu_{\hat{m}}=\underset{\hat{m}\in \Omega \xi }{\sum }\left(\begin{array}{c} \bar{m}\\ \hat{m} \end{array}\right){\vec{\omega }}^{\hat{m}}$$

First, we focus on the left hand side of the above equation. From (37), and (22) with $\vec{m}={\hat{m}}_{1}\in \Omega_{\xi }$,

(39) $$\ln\;\nu_{\hat{m}1}=\underset{m2\in \Omega \xi }{\sum }\left(\begin{array}{c} {\hat{m}}_{1}\\ {\hat{m}}_{2} \end{array}\right){\vec{\omega }}^{{\hat{m}}_{2}}$$

which by the use of the binomial theorem can be recognized as

(40) $$\ln\;\nu_{\hat{m}}={\left(1+\vec{\omega }\right)}^{\hat{m}}$$

This turns the left hand side of (38) into

(41) $$\underset{\hat{m}\in \Omega_{\xi }}{\sum }\gamma_{\hat{m}}^{\bar{m}}\ln\;\nu_{\hat{m}}=\underset{\hat{m}\in \Omega \xi }{\sum }\gamma_{\hat{m}}^{\bar{m}}{\left(1+\vec{\omega }\right)}^{\hat{m}}$$

The sum over the vectors in Ω**_ξ _**in the right hand side of (38) can be extended to include all vectors in $\Omega_{\bar{m}}$. Naturally, these terms have to be subtracted afterwards. By doing that one obtains

(42) $$\underset{\hat{m}\in \Omega_{\xi }}{\sum }\left(\begin{array}{c} \bar{m}\\ \hat{m} \end{array}\right){\vec{\omega }}^{\hat{m}}=\underset{\vec{m}\in \Omega_{\bar{m}}}{\sum }\left(\begin{array}{c} \bar{m}\\ \vec{m} \end{array}\right){\vec{\omega }}^{\vec{m}}-\underset{{\bar{m}}^{\prime }\in \Omega_{\bar{m}}\backslash \Omega \xi }{\sum }\left(\begin{array}{c} \bar{m}\\ {\bar{m}}^{\prime } \end{array}\right){\vec{\omega }}^{{\bar{m}}^{\prime }}$$

By the use of the binomial theorem the first term on the right hand side of the equation can be recognized as the first term in the right hand of (34). Likewise, it is trivial to see that the second term in the equation can be characterized as $P_{\xi +1}\left(\vec{\omega }\right)$. Thus the equation above becomes,

(43) $$\underset{\hat{m}\in \Omega_{\xi }}{\sum }\left(\begin{array}{c} \bar{m}\\ \hat{m} \end{array}\right){\vec{\omega }}^{\hat{m}}={\left(1+\vec{\omega }\right)}^{\bar{m}}+P_{\xi +1}\left(\vec{\omega }\right)$$

Finally, the Lemma follows by using (38), (41), and (43).

A couple of useful identities will be proven that follow from this Lemma and are stated as three corollaries. The first two corollaries can be proven easily without using the Lemma, e.g. as in [10,11]. In here they are proven in a different way to illustrate how to use the Lemma. The third corollary is a highly non trivial statement that would be very hard to prove by direct use of the explicit form of *γ *coefficients.

**Corollary 1**. Let coefficients *γ *be given and let them satisfy the condition of Lemma 1. Then,

(44) $$\underset{{\hat{m}}_{2}\in \Omega_{\xi }}{\sum }\gamma_{{\hat{m}}_{2}}^{\bar{m}}\left(\begin{array}{c} {\hat{m}}_{2}\\ {\hat{m}}_{1} \end{array}\right)=\left(\begin{array}{c} \bar{m}\\ {\hat{m}}_{1} \end{array}\right);{\hat{m}}_{1}\in \Omega_{\xi }$$

*Proof*. The proof is trivial. One only needs to apply the operator $\left[{\vec{\omega }}^{{\hat{m}}_{1}}\right]$ on the both sides of the generating function equation (34).

**Corollary 2**. Let coefficients *γ *be given and let them satisfy the condition of Lemma 1. Then,

(45) $$\underset{\hat{m}\in \Omega_{\xi }}{\sum }\hat{m}\gamma_{\hat{m}}^{\bar{m}}=\bar{m}$$

*Proof*. The identity can be obtained by evaluating the gradient of Eq. (34) with respect to $\vec{\omega }$ at the point $\vec{\omega }=\vec{0}$.

**Corollary 3**. Let the coefficients *γ *satisfy the generating function equation (34). Then following identity holds

(46) $$\underset{\hat{m}\in \Omega_{\xi }}{\sum }\gamma_{\hat{m}}^{\bar{m}}\left(\begin{array}{c} \hat{m}\\ {\vec{c}}_{1} \end{array}\right)\left(\begin{array}{c} \hat{m}\\ {\vec{c}}_{2} \end{array}\right)=\left(\begin{array}{c} \bar{m}\\ {\vec{c}}_{1} \end{array}\right)\left(\begin{array}{c} \bar{m}\\ {\vec{c}}_{2} \end{array}\right)$$

provided vectors ${\vec{c}}_{1}$ and ${\vec{c}}_{2}$ satisfy $\left|{\vec{c}}_{1}+{\vec{c}}_{2}\right|\leq \xi$.

*Proof*. First, one has to assume that

(47) $$\vec{\omega }={\vec{\omega }}_{1}+{\vec{\omega }}_{2}+{\vec{\omega }}_{1}⊙{\vec{\omega }}_{2}⊙$$

where ${\vec{\omega }}_{1},{\vec{\omega }}_{2}\in R^{T}$ are arbitrary but bound by the above constraint. Also, it is useful to realize that

(48) $$1+\vec{\omega }=1+{\vec{\omega }}_{1}+{\vec{\omega }}_{2}+{\vec{\omega }}_{1}⊙{\vec{\omega }}_{2}=\left(1+{\vec{\omega }}_{1}\right)⊙\left(1+{\vec{\omega }}_{2}\right)$$

By using the above expression in the generating function formula, and (17), one obtains

(49) $$\begin{array}{c} \underset{\hat{m}\in \Omega \xi }{\sum }\gamma_{\hat{m}}^{\bar{m}}{\left(1+{\vec{\omega }}_{1}\right)}^{\hat{m}}{\left(1+{\vec{\omega }}_{2}\right)}^{\hat{m}}=\\ \;\;\;\;\;{\left(1+{\vec{\omega }}_{1}\right)}^{\bar{m}}{\left(1+{\vec{\omega }}_{2}\right)}^{\bar{m}}+\\ \;\;P_{\xi +1}\left({\vec{\omega }}_{1}+{\vec{\omega }}_{2}+{\vec{\omega }}_{1}⊙{\vec{\omega }}_{2}\right) \end{array}$$

In the third step one has to apply operators $\left[{\vec{\omega }}_{1}^{{\vec{c}}_{1}}\right]$ and $\left[{\vec{\omega }}_{1}^{{\vec{c}}_{2}}\right]$ to the generating function expression above. Applying the operators to the left hand side of the equation above gives the left hand side of Eq. (46). Likewise by applying the operators to the first term on the right hand side of the equation gives the right hand side of Eq. (46). Thus what is left to show is that the action of the operator onthe remaining second term results in zero.

The second term has the following structure

(50) $$\begin{array}{c} P_{\xi +1}\left({\vec{\omega }}_{1}+{\vec{\omega }}_{2}+{\vec{\omega }}_{1}⊙{\vec{\omega }}_{2}\right)=\\ \overset{∥\vec{p}+\vec{q}+\vec{s}∥\geq \xi +1}{\underset{\vec{p},\vec{q},\vec{s}\in \Omega }{\sum }}A_{\vec{p},\vec{q},\vec{s}}{\vec{\omega }}_{1}^{\vec{p}}{\vec{\omega }}_{1}^{\vec{q}}{\left({\vec{\omega }}_{1}⊙{\vec{\omega }}_{2}\right)}^{\vec{s}} \end{array}$$

where *A *coefficients can be found but their exact form is not relevant. Next, by using (17) and (13) the equation becomes

(51) $$\begin{array}{c} P_{\xi +1}\left({\vec{\omega }}_{1}+{\vec{\omega }}_{2}+{\vec{\omega }}_{1}⊙{\vec{\omega }}_{2}\right)=\\ \overset{∥\vec{p}+\vec{q}+\vec{s}∥\geq \xi +1}{\underset{\vec{p},\vec{q},\vec{s}\in \Omega }{\sum }}A_{\vec{p},\vec{q},\vec{s}}{\vec{\omega }}_{1}^{\vec{p}+\vec{s}}{\vec{\omega }}_{2}^{\vec{q}+\vec{s}} \end{array}$$

and by a simple change of variables $\vec{p}+\vec{s}={\vec{c}}_{1}$ and $\vec{q}+\vec{s}={\vec{c}}_{2}$ one arrives at

(52) $$\begin{array}{c} P_{\xi +1}\left({\vec{\omega }}_{1}+{\vec{\omega }}_{2}+{\vec{\omega }}_{1}⊙{\vec{\omega }}_{2}\right)=\\ \overset{∥{\vec{c}}_{1}+{\vec{c}}_{2}∥\geq \xi +1}{\underset{{\vec{c}}_{1},{\vec{c}}_{2},\in \Omega }{\sum }}A_{{\vec{c}}_{1},{\vec{c}}_{2}}{\vec{\omega }}_{1}^{{\vec{c}}_{1}}{\vec{\omega }}_{2}^{{\vec{c}}_{2}} \end{array}$$

To obtain the final form of the equation, and in particular the condition specified in the sum, one has to use the fact that $∥{\vec{c}}_{1}+{\vec{c}}_{2}∥=∥\vec{p}+\vec{q}+2\vec{s}∥\geq ∥\vec{p}+\vec{q}+\vec{s}∥\geq \xi +1$. Finally, from the last equation one can see that, indeed, the application of the operator $\left[{\vec{\omega }}_{1}^{{\vec{c}}_{1}}\right]\left[{\vec{\omega }}_{2}^{{\vec{c}}_{2}}\right]$ gives zero provided that $∥{\vec{c}}_{1}+{\vec{c}}_{2}∥\leq \xi$. This proves the corollary. Please note that it would be very hard, if not impossible, to obtain such a result from the explicit expression for *γ *coefficients given in (25).

Higher order generalizations of this corollary are possible. For example, by using

(53) $$1+\vec{\omega }=\left(1+{\vec{\omega }}_{1}\right)⊙\left(1+{\vec{\omega }}_{2}\right)⊙\left(1+{\vec{\omega }}_{3}\right)$$

one can easily prove that

(54) $$\underset{\hat{m}\in \Omega_{\xi }}{\sum }\gamma_{\hat{m}}^{\bar{m}}\left(\begin{array}{c} \hat{m}\\ {\vec{c}}_{1} \end{array}\right)\left(\begin{array}{c} \hat{m}\\ {\vec{c}}_{2} \end{array}\right)\left(\begin{array}{c} \hat{m}\\ {\vec{c}}_{3} \end{array}\right)=\left(\begin{array}{c} \bar{m}\\ {\vec{c}}_{1} \end{array}\right)\left(\begin{array}{c} \bar{m}\\ {\vec{c}}_{2} \end{array}\right)\left(\begin{array}{c} \bar{m}\\ {\vec{c}}_{3} \end{array}\right)$$

provided that vectors ${\vec{c}}_{1}$, ${\vec{c}}_{2}$, and ${\vec{c}}_{3}$ satisfy $∥{\vec{c}}_{1}+{\vec{c}}_{2}+{\vec{c}}_{3}∥\leq \xi$.

Now we are ready to prove some derivative matching results. The first question is whether the XARNES ansatz and the related moment closure function are consistent for the Poisson initial condition. This is answered in a form of the following Lemma.

**Lemma 2**. If the base and the exact factorial moments match at *t = t*_0_, and the particle number distribution function at this time instance is the Poisson distribution, i.e.

(55) $$\rho_{\hat{m}}\left(t_{0}\right)=\nu_{\hat{m}}\left(t_{0}\right)={\vec{\mu }}^{\hat{m}};\hat{m}\in \Omega \xi$$

then non-base moments also match:

(56) $$\rho_{\hat{m}}\left(t_{0}\right)=\nu_{\hat{m}}\left(t_{0}\right)=\psi_{\bar{m}}\left(v\left(t_{0}\right)\right)={\vec{\mu }}^{\bar{m}}$$

for all $\bar{m}\in {\bar{\Omega }}_{\xi }$.

*Proof*. The direct use of the XARNES ansatz gives

(57) $$\ln\;\nu_{\bar{m}}=\underset{\hat{m}\in \Omega \xi }{\sum }\hat{m}\gamma_{\hat{m}}^{\bar{m}}\ln\;\vec{\mu }=\bar{m}\ln\;\vec{\mu }$$

where (45) was used in the last step.

Finally, we arrive at the central discussion of this work, i.e. the discussion of various time derivatives of exact and approximate moments and when and how they differ. For doing this it is instructive to investigate the difference between the exact and the XARNES equation systems. In this context one can easily show that the following equation system is valid,

(58) $$\begin{array}{c} p_{\hat{m}}^{\left[h\right]}-v_{\hat{m}}^{\left[h\right]}=\overset{R}{\underset{\alpha =1}{\sum }}\lambda_{\alpha }\underset{\vec{c}\in \Omega R}{\sum }\left(\begin{array}{c} \hat{m}\\ \vec{c} \end{array}\right)\;\Gamma_{\alpha }\left(\vec{c}\right)\times \\ \left\{\underset{{\hat{m}}_{1}\in \Omega \xi }{\sum }\left[\rho_{{\hat{m}}_{1}}^{\left[h-1\right]}-\psi_{{\hat{m}}_{1}}^{\left[h-1\right]}\right]\delta_{{\hat{m}}_{1},\hat{m}-\vec{c}+\vec{u}\alpha }+\right)\\ \left(\underset{\hat{m}\in \Omega \xi }{\sum }\left[\rho_{\hat{m}}^{\left[h-1\right]}-\psi_{\hat{m}}^{\left[h-1\right]}\right]\delta_{\hat{m},\hat{m}-\vec{c}+\vec{u}\alpha }\right\} \end{array}$$

and notation $\varphi^{\left[h\right]}\equiv \frac{d^{h}}{dt^{h}}\varphi \left(t\right){|}_{t=t_{0}}$ with *h *= 1,2,3,... is implied where *φ(t) *is any function, and *φ*^[0] ^≡ *φ*(*t*_0_). The equation system above will be useful for proving a series of derivative matching theorems, in the similar vein as done in [10,11].

## Three derivative matching theorems

Three derivative matching theorems will be proven, one theorem per derivative. The first two theorems have been proven in [10,11] for the pure state. In here they are proven for the Poisson state. The third theorem is entirely new.

The structure of the proofs is somewhat different than in [10,11] since in here the focus is on factorial moments. It seems that the equation system for factorial moments is more compact than for other types of moments. As an artifact of that, the theorems do not contain the error terms *ε *that were used in [10,11]. The theorems proven in here are more generic since they hold even for multi particle reactions, not just binary reactions. Again, as stated previously, all components of $\vec{\mu }$ are taken strictly larger than zero. If one of the components is zero the XARNES ansatz does not work.

**Theorem 1**. If the base and the exact factorial moments match at *t = t*_0_, and the particle number distribution function is the Poisson distribution, i.e., if

(59) $$\rho_{\hat{m}}\left(t_{0}\right)=v_{\hat{m}}\left(t_{0}\right)={\vec{\mu }}^{\hat{m}};\hat{m}\in \Omega_{\xi }$$

then the first order derivatives in time also match for the base factorial moments:

(60) $$\frac{d}{dt}\rho_{\hat{m}}\left(t\right){|}_{t=t_{0}}=\frac{d}{dt}\nu_{\hat{m}}\left(t\right){|}_{t=t_{0}}$$

for all $\hat{m}\in \Omega_{\xi }$.

*Proof*. The theorem can be easily proven by considering Eq. (58) with *h = *1. By assumptions of the theorem one has that $p_{\hat{m}}^{\left[0\right]}-\psi_{\hat{m}}^{\left[0\right]}=0$ which eliminates the sum over $\hat{m}\in \Omega_{\xi }$ in (58). From Lemma 2 it follows that $\rho_{\bar{m}}^{\left[0\right]}-\psi_{\bar{m}}^{\left[0\right]}=0$ which eliminates the sum over $\bar{m}\in {\bar{\Omega }}_{\xi }$. This finally proves the theorem.

**Theorem 2**. If the base and the exact factorial moments match at *t = t*_0_, and the particle number distribution function is the Poisson distribution, i.e., if

(61) $$\rho_{\hat{m}}\left(t_{0}\right)=\nu_{\hat{m}}\left(t_{0}\right)={\vec{\mu }}^{\hat{m}};\hat{m}\in \Omega_{\xi }$$

and if Ω*_R _*⊂ Ω*_ξ_*, then the second order derivatives in time also match:

(62) $$\frac{d^{2}}{dt^{2}}\rho_{\hat{m}}\left(t\right){|}_{t=t_{0}}=\frac{d^{2}}{dt^{2}}v_{\hat{m}}\left(t\right){|}_{t=t_{0}};\hat{m}\in \Omega_{\xi }$$

*Proof*. The proof of this theorem is somewhat lengthier. To prove the theorem one needs to consider Eq. (58) with *h *= 2. If the assumptions of the theorem are valid, by theorem 1, $p_{\hat{m}}^{\left[1\right]}-\psi_{\hat{m}}^{\left[1\right]}=0$ which eliminates the sum over $\hat{m}\in \Omega_{\xi }$ in (58). Thus what is left to show is that a difference $\rho_{\bar{m}}^{\left[1\right]}-\psi_{\bar{m}}^{\left[1\right]}$ with $\bar{m}\in r\Omega_{\xi }$ vanishes.

A straight forward application of the time derivative on the moment closure function leads to the following expression,

(63) $$\psi_{\bar{m}}^{\left[1\right]}=\underset{\hat{m}\in \Omega_{\xi }}{\sum }\frac{\partial \psi \bar{m}}{\partial \psi \hat{m}}v_{\hat{m}}^{\left[1\right]}=\underset{\hat{m}\in \Omega_{\xi }}{\sum }\gamma_{\hat{m}}^{\bar{m}}{\vec{\mu }}^{\bar{m}-\hat{m}}v_{\hat{m}}^{\left[1\right]}$$

Also, the use of the exact, and the XARNES equation systems to evaluate $\;\rho_{\bar{m}}^{\left[1\right]}$ and $\psi_{\bar{m}}^{\left[1\right]}$ gives

(64) $$\begin{array}{c} \rho_{\bar{m}}^{\left[1\right]}-\psi_{\bar{m}}^{\left[1\right]}=\underset{\vec{c}\in \Omega_{R}}{\sum }\left[\left(\begin{array}{c} \bar{m}\\ \vec{c} \end{array}\right)-\underset{\;\hat{m}\in \Omega_{\xi }}{\sum }\gamma_{\hat{m}}^{\bar{m}}\left(\begin{array}{c} \hat{m}\\ \vec{c} \end{array}\right)\right]\times \\ \overset{R}{\underset{\alpha =1}{\sum }}\lambda_{\alpha }\Gamma_{\alpha }\left(\vec{c}\right){\vec{\mu }}^{\bar{m}+{\vec{u}}_{\alpha }-\vec{c}} \end{array}$$

This difference is zero provided

(65) $$\underset{\hat{m}\in \Omega_{\xi }}{\sum }\gamma_{\hat{m}}^{\bar{m}}\left(\begin{array}{c} \hat{m}\\ \vec{c} \end{array}\right)=\left(\begin{array}{c} \bar{m}\\ \vec{c} \end{array}\right)$$

for every $\vec{c}\in \Omega_{R}$. Please note that this condition is almost identical to the equation that characterize the *γ *coefficients of the XARNES ansatz. In one replaces $\vec{c}$ with $\hat{m}$, and Ω*_R _*with Ω*_ξ _*in the equation above, then the equation obtained in such a way would be identical to Eq. (25) or (44). Thus if Ω*_R _*⊂ Ω*_ξ _*then the equation above is contained in the condition that defines the *γ *coefficients, and the equation is automatically valid. This proves the theorem.

The third order derivatives will be investigated in the same vein as the first and the second order derivatives. The result will be formulated in a precise mathematical theorem. However, before stating the next theorem, it is useful to generalize the space of contraction vectors as follows.

**Definition 12**. Vector space of sums of contraction vectors ${\vec{c}}_{1}+{\vec{c}}_{2}+\cdots +{\vec{c}}_{h}$ where each of the vectors in the sum is from Ω*_R_*, will be denoted as

(66) $$\Omega_{h⊗R}=\left\{{\vec{c}}_{1}+{\vec{c}}_{2}+\cdots +{\vec{c}}_{h}|{\vec{c}}_{1},{\vec{c}}_{2}\cdots \;,{\vec{c}}_{h}\in \Omega_{R}\right\}$$

and *h *is an integer and obeys *h *≥ 1.

**Theorem 3**. If the base and the exact factorial moments match at *t *= *t*_0 _and the particle number distribution function is the Poisson distribution, i.e., if

(67) $$\rho_{\hat{m}}\left(t_{0}\right)=\nu_{\hat{m}}\left(t_{0}\right)={\vec{\mu }}^{\hat{m}};\hat{m}\in \Omega_{\xi }$$

and if *Ω*_2⊗*R *_⊂ *Ω_ξ_*, then the third order derivatives in time also match

(68) $$\frac{d^{3}}{dt^{3}}\rho_{\hat{m}}\left(t\right){|}_{t=t_{0}}=\frac{d^{3}}{dt^{3}}\nu_{\hat{m}}\left(t\right){|}_{t=t_{0}};\hat{m}\in \Omega_{\xi }$$

*Proof*. The theorem can be proven by considering Eq. (58) with *h *= 3. By theorem 2 one has that $\rho_{\hat{m}}^{\left[2\right]}-\psi_{\hat{m}}^{\left[2\right]}=0$. What is left to show is that all differences $\rho_{\bar{m}-\psi_{\bar{m}}^{\left[2\right]}}^{\left[2\right]}$ with $\bar{m}\in {\bar{\Omega }}_{\xi }$ vanish as well. Unfortunately this is a highly nontrivial task.

By the use of the standard calculus one can show that

(69) $$\psi_{\bar{m}}^{\left[2\right]}=\psi_{\bar{m},†}^{\left[2\right]}+\psi_{\bar{m},‡}^{\left[2\right]}$$

where

(70) $$\begin{array}{c} \psi_{\bar{m},†}^{\left[2\right]}=\underset{{\hat{m}}_{1},{\hat{m}}_{2}\in \Omega_{\xi }}{\sum }\left(\gamma_{{\hat{m}}_{1}}^{\bar{m}\gamma }\gamma_{{\hat{m}}_{2}}^{\bar{m}\gamma }-\gamma_{{\hat{m}}_{1}}^{\bar{m}\gamma }\delta_{{\hat{m}}_{1},{\hat{m}}_{2}}\right)\times \\ \;\;\;\;\;\;\mu^{\bar{m}-{\hat{m}}_{1}-{\hat{m}}_{2}}v_{{\hat{m}}_{1}}^{\left[1\right]}v_{{\hat{m}}_{2}}^{\left[1\right]} \end{array}$$

and

(71) $$\psi_{\bar{m},‡}^{\left[2\right]}=\underset{\hat{m}\in \Omega_{\xi }}{\sum }\gamma_{\hat{m}}^{\bar{m}}{\vec{\mu }}^{\bar{m}-\hat{m}}\nu_{\hat{m}}^{\left[2\right]}$$

By using the XARNES equations, and identity (44) for *γ *coefficients, the $\psi_{\bar{m},†}^{\left[2\right]}$ can be expressed as

(72) $$\begin{array}{c} \psi_{\bar{m},†}^{\left[2\right]}=,\;\underset{{\vec{c}}_{1},{\vec{c}}_{2}\in \Omega_{R}}{\sum }\left[\left(\begin{array}{c} \bar{m}\\ {\vec{c}}_{1} \end{array}\right)\left(\begin{array}{c} \bar{m}\\ {\vec{c}}_{2} \end{array}\right)-\underset{{}_{\hat{m}\in \Omega \xi }}{\sum }\gamma_{\hat{m}}^{\bar{m}}\left(\begin{array}{c} \hat{m}\\ {\vec{c}}_{1} \end{array}\right)\left(\begin{array}{c} \hat{m}\\ {\vec{c}}_{2} \end{array}\right)\right]\times \\ \;\underset{\alpha ,\beta }{\sum }\lambda_{\alpha }\lambda_{\beta }\Gamma_{\alpha }\left({\vec{c}}_{1}\right)\Gamma_{\beta }\left({\vec{c}}_{2}\right){\vec{\mu }}^{\bar{m}+\vec{u}\alpha +{\vec{u}}_{\beta }-{\vec{c}}_{1}-{\vec{c}}_{2}} \end{array}$$

which vanishes provided

(73) $$\underset{\hat{m}\in \Omega_{\xi }}{\sum }\gamma_{\hat{m}}^{\bar{m}}\left(\begin{array}{c} \hat{m}\\ {\vec{c}}_{1} \end{array}\right)\left(\begin{array}{c} \hat{m}\\ {\vec{c}}_{2} \end{array}\right)=\left(\begin{array}{c} \bar{m}\\ {\vec{c}}_{1} \end{array}\right)\left(\begin{array}{c} \bar{m}\\ {\vec{c}}_{2} \end{array}\right)$$

for any ${\vec{c}}_{1},{\vec{c}}_{2}\in \Omega_{R}$. This is indeed true by corollary 3 under the assumptions of the theorem.

What is left to show is that $\rho_{\bar{m}}^{\left[2\right]}-\psi_{\bar{m},‡}^{\left[2\right]}=0$. A strategy for proving this is as follows. If one could show that the following identity holds for the exact moments

(74) $$\Delta_{\bar{m}}\equiv \rho_{\bar{m}}^{\left[2\right]}-\underset{\hat{m}\in \Omega \xi }{\sum }\gamma_{\hat{m}}^{\bar{m}}{\vec{\mu }}^{\bar{m}-\hat{m}}\rho_{\hat{m}}^{\left[2\right]}=0$$

then it is clear that $\rho_{\bar{m}}^{\left[2\right]}-\psi_{\bar{m},‡}^{\left[2\right]}=0$ would be true since one could write

(75) $$\rho_{\bar{m}}^{\left[2\right]}-\psi_{\bar{m},‡}^{\left[2\right]}=\underset{\hat{m}\in \Omega \xi }{\sum }\gamma_{\hat{m}}^{\bar{m}}{\vec{\mu }}^{\bar{m}-\hat{m}}\left(\rho_{\hat{m}}^{\left[2\right]}-\psi_{\hat{m}}^{\left[2\right]}\right)$$

and this expression would vanish by Theorem 2.

Somewhat naive recursive application of the exact equations of motion results in

(76) $$\begin{array}{c} \rho_{\bar{m}}^{\left[2\right]}=\underset{\alpha ,\beta }{\sum }\lambda_{\alpha }\lambda_{\beta }\underset{{\vec{c}}_{1},{\vec{c}}_{2}\in \Omega_{R}}{\sum }\left(\begin{array}{c} \bar{m}\\ {\vec{c}}_{1} \end{array}\right)\left(\begin{array}{c} \bar{m}+{\vec{u}}_{\alpha }-{\vec{c}}_{1}\\ {\vec{c}}_{2} \end{array}\right)\times \\ \Gamma_{\alpha }\left({\vec{c}}_{1}\right)\Gamma_{\beta }\left({\vec{c}}_{2}\right){\vec{\mu }}^{\bar{m}+{\vec{u}}_{\alpha }-{\vec{u}}_{\beta }-{\vec{c}}_{1}-{\vec{c}}_{2}}\; \end{array}$$

By using a tedious manipulation of the binomial coefficients the expression above can be converted into a more useful form

(77) $$\begin{array}{c} \rho_{\bar{m}}^{\left[2\right]}=\underset{\alpha ,\beta }{\sum }\lambda_{\alpha }\lambda_{\beta }\underset{{\vec{c}}_{1},{\vec{c}}_{2},\vec{d}\in \Omega_{R}}{\sum }\left(\begin{array}{c} \bar{m}\\ c_{1} \end{array}\right)\left(\begin{array}{c} \bar{m}-{\vec{c}}_{1}\\ c_{2} \end{array}\right)\times \\ \left(\frac{{\vec{u}}_{\alpha }}{d}\right)\Gamma_{\alpha }\left({\vec{c}}_{1}\right)\Gamma_{\beta }\left({\vec{c}}_{2}+\vec{d}\right){\vec{\mu }}^{\bar{m}+{\vec{u}}_{\alpha }+{\vec{u}}_{\beta }-{\vec{c}}_{1}-{\vec{c}}_{2}-\vec{d}} \end{array}$$

In fact, it is easier to start from (77) and obtain (76). First, the manipulation requires that the sum of ${\vec{c}}_{2}$ and $\vec{d}$ is changed, into the sum over ${\vec{c}}_{2}={\vec{c}}_{2}+\vec{d}$ and *${\vec{c}}_{3}={\vec{c}}_{2}$*. After that the Vandermonde identity needs to be used which consumes the sum over ${\vec{c}}_{3^{\prime }}$, resulting finally in (77).

By using the fact that

(78) $$\left(\begin{array}{c} \bar{m}\\ {\vec{c}}_{1} \end{array}\right)\left(\begin{array}{c} \bar{m}-{\vec{c}}_{1}\\ {\vec{c}}_{2} \end{array}\right)=\left(\begin{array}{c} \bar{m}\\ {\vec{c}}_{1}+{\vec{c}}_{2} \end{array}\right)\left(\begin{array}{c} {\vec{c}}_{1}+{\vec{c}}_{2}\\ {\vec{c}}_{2} \end{array}\right)$$

Eq (77) can be written in the most useful form as

(79) $$\begin{array}{c} \;\rho_{\bar{m}}^{\left[2\right]}=\underset{{\vec{c}}_{1},{\vec{c}}_{2}\in \Omega_{R}}{\sum }\left(\begin{array}{c} \bar{m}\\ {\vec{c}}_{1}+{\vec{c}}_{2} \end{array}\right)\times \\ \underset{\alpha ,\beta }{\sum }\Lambda_{\alpha }{,}_{\beta }\left(\vec{\mu },{\vec{c}}_{1},{\vec{c}}_{2}\right){\vec{\mu }}^{\bar{m}+{\vec{u}}_{\alpha }+{\vec{u}}_{\beta }-{\vec{c}}_{1}-{\vec{c}}_{2}} \end{array}$$

The exact form of a coefficient $\Lambda_{\alpha ,\beta }\left(\vec{\mu },{\vec{c}}_{1},{\vec{c}}_{2}\right)$ can be found if needed and, in fact, it is a series in $\vec{\mu }$. However, the exact form of these coefficients is not relevant for the discussion that follows.

Let us use (79) in (74). This gives

(80) $$\begin{array}{c} \Delta_{\bar{m}}=\underset{{\vec{c}}_{1},{\vec{c}}_{2}\in \Omega_{R}}{\sum }\left[\left(\begin{array}{c} \bar{m}\\ {\vec{c}}_{1}+{\vec{c}}_{2} \end{array}\right)-\underset{{\hat{m}}_{1}\in \Omega_{\xi }}{\sum }\gamma_{{\hat{m}}_{1}}^{\bar{m}}\left(\begin{array}{c} {\hat{m}}_{1}\\ {\vec{c}}_{1}+{\vec{c}}_{2} \end{array}\right)\right]\times \\ \underset{\alpha ,\beta }{\sum }\Lambda_{\alpha ,\beta }\left(\vec{\mu },{\vec{c}}_{1},{\vec{c}}_{2}\right){\vec{\mu }}^{\bar{m}+{\vec{u}}_{\alpha }+{\vec{u}}_{\beta }-{\vec{c}}_{1}-{\vec{c}}_{2}} \end{array}$$

and $\Delta_{\bar{m}}$ is zero if the following condition is met,

(81) $$\underset{\hat{m}\in \Omega_{\xi }}{\sum }\gamma_{\hat{m}}^{\bar{m}}\left(\begin{array}{c} \hat{m}\\ {\vec{c}}_{1,2} \end{array}\right)=\left(\begin{array}{c} \bar{m}\\ {\vec{c}}_{1,2} \end{array}\right)$$

for every ${\vec{c}}_{1,2}\in \Omega_{2⊗R}$. Eq. (81) is satisfied by the assumption of the theorem which states that *Ω*_2⊗*R *_⊂ *Ω_ξ_*. In such a case equations in (81) are a subset of the equations satisfied by the *γ *coefficients which are given in (44) and are automatically valid.

The three theorems proven so far are suggestive of the fact that one might try to prove the following conjecture.

**Conjecture 1. If **the base and the exact factorial moments match at *t *= *t*_0_, and the particle number distribution function is the Poisson distribution, i.e., if

(82) $$\rho_{\hat{m}}\left(t_{0}\right)=\nu_{\hat{m}}\left(t_{0}\right)={\vec{\mu }}^{\hat{m}};\hat{m}\in \Omega_{\xi }$$

and if *Ω*_*h*⊗*R *_⊂ *Ω_ξ_*, where *h *is an arbitrary integer such that *h *≥ 1, then the time derivatives with orders *D *= 0, 1, 2, **...**, *h *+ 1 will also match

(83) $$\frac{d^{D}}{dt^{D}}\rho_{\hat{m}}\left(t\right){|}_{t=t_{0}}=\frac{d^{D}}{dt^{D}}v_{\hat{m}}\left(t\right){|}_{t=t_{0}};\hat{m}\in \Omega_{\xi }$$

*Eventual. Inductive *proof for general *h *could be used. However, the problems is that one would need to inspect a difference $\rho_{\bar{m}}^{\left[h\right]}-\psi_{\bar{m}}^{\left[h\right]}$ for arbitrary *h *and show that it vanishes under some conditions. Presumably, the main condition, apart from the standard requirements, e.g. such as having the Poisson initial condition, would be that *Ω*_*h*⊗*R *_⊂ *Ω_ξ_*. For example, one can easily see that an expressions such as the one shown in (53) will appear if one tries to calculate higher time derivatives of $\psi_{\bar{m}}$. However, as demonstrated for the *h *= 2 (*D *= 3) case the computation of $\psi_{\bar{m}}^{\left[h\right]}$ for higher values of *h *is a rather cumbersome and technical procedure. Generalization to higher orders is apparently very hard but not impossible.

There are couple of reasons why such a conjecture might be valid. First, the structure of the proofs of theorems 1-3 (the cases *h *= 0, 1, 2) suggests such a possibility. Second, there is numerical evidence from a previous study [17] that increase in *ξ *improves the accuracy of the XARNES method. In the context of the theorems discussed in here, increase in *ξ *implies that the initial *Ω_ξ _*set becomes larger. This in turncan make the condition *Ω*_*h*⊗*R *_⊂ *Ω_ξ _*valid for a larger value of *h*. Finally as a result of that a larger number of derivatives would match which would explain the observed accuracy improvements in the studied benchmark cases. Finally, third, this conjecture has be checked by Mathematica for the *T *= 1 case and two binary reactions 2*X*_1 _→ 0 and 2*X*_1 _→ *X*_1_, both with *h *= 0, 1, 2, 3, 4, 5 and *ξ *= 2, 4, 6, 8, 10 respectively, and one multi particle reaction 3*X*_1 _→ 2*X*_1 _with *h *= 0, 1, 2, 3 and *ξ *= 3, 6, 9.

## Conclusions

The three theorems explain the mechanism behind the numerically observed fact that the XARNES method works well if the particle number distribution function is close to the Poisson distribution. Thus if all correlation forms are in some sense small, the XARNES method will provide a very accurate result.

For very small molecule counts one should expect problems with moment closure formulas like (24) with negative *γ *coefficients. The XARNES ansatz can describe such situation but one needs to consider a limiting processwhere factorial moments approach zero from the above. For example, one might want to start the systems from a state with the Poisson distribution, which is natural in many biologically relevant cases, but with some components of $\vec{\mu }$ equal to zero. This cannot be done directly since the XARNES ansatz breaks down. In more practical terms, any decent numerical Ordinary Differential Equation (ODE) solver should issue a warning for such an initial state. To start the system from a state wheresome copy numbers are zero it is necessary to consider increasingly smaller values for such copy numbers.

Previous numerical studies showed that there are systems for which the XARNES ODE system develops a singularity and the numerical solver has to stop [14,15,17]. Unfortunately, the theorems that have been proven cannot say anything about such singularities since the particle number distribution function becomes increasingly different from the Poisson distribution.

## Competing interests

The authors declare that they have no competing interests.
